# An Adaptive Shrinking Grid Search Chaotic Wolf Optimization Algorithm Using Standard Deviation Updating Amount

**DOI:** 10.1155/2020/7986982

**Published:** 2020-05-18

**Authors:** Dongxing Wang, Xiaojuan Ban, Linhong Ji, Xinyu Guan, Kang Liu, Xu Qian

**Affiliations:** ^1^Beijing Advanced Innovation Center for Materials Genome Engineering, University of Science &Technology Beijing, Beijing 100083, China; ^2^School of Mechanical Electronic & Information Engineering, China University of Mining & Technology-Beijing, Beijing 100083, China; ^3^Department of Mechanical Engineering, Tsinghua University, Beijing 100084, China

## Abstract

To improve the optimization quality, stability, and speed of convergence of wolf pack algorithm, an adaptive shrinking grid search chaotic wolf optimization algorithm using standard deviation updating amount (ASGS-CWOA) was proposed. First of all, a strategy of adaptive shrinking grid search (ASGS) was designed for wolf pack algorithm to enhance its searching capability through which all wolves in the pack are allowed to compete as the leader wolf in order to improve the probability of finding the global optimization. Furthermore, opposite-middle raid method (OMR) is used in the wolf pack algorithm to accelerate its convergence rate. Finally, “Standard Deviation Updating Amount” (SDUA) is adopted for the process of population regeneration, aimed at enhancing biodiversity of the population. The experimental results indicate that compared with traditional genetic algorithm (GA), particle swarm optimization (PSO), leading wolf pack algorithm (LWPS), and chaos wolf optimization algorithm (CWOA), ASGS-CWOA has a faster convergence speed, better global search accuracy, and high robustness under the same conditions.

## 1. Introduction

### 1.1. Literature Review

The metaheuristic search technology based on swarm intelligence has been increasing in popularity due to its ability to solve a variety of complex scientific and engineering problems [1]. The technology models the social behavior of certain living creatures, in which each individual is simple and has limited cognitive capability, but the swarm can act in a coordinated way without a coordinator or an external commander and yield intelligent behavior to obtain global optima as a whole [2]. In [3], Yang Cuicui et al. adopted bacterial foraging optimization to optimize the structural learning of Bayesian networks. In [4] and [5], swarm intelligent algorithm is used for functional module detection in protein-protein interaction networks to help biologists to find some novel biological insights. In [6], Ji et al. performed a systematic comparison of three typical methods based on ant colony optimization, artificial bee colony algorithm, and bacterial foraging optimization regarding how to accurately and robustly learn a network structure for a complex system. In [7], the authors utilize the artificial immune algorithm to infer the effective connectivity between different brain regions. In [8], researchers used an ant colony optimization algorithm for learning brain effective connectivity network from fMRI data. Particle swarm optimization (PSO) [9] algorithm was proposed through the observation and study of the bird group's flapping behavior. Ant colony optimization (ACO) [10] algorithm was proposed under the principle of simulating ant social division of labor and cooperative foraging. Fish swarm algorithm (FSA) [11] was proposed to simulate the behavior of foraging and clustering in the fish group. In [12], bacterial foraging optimization (BFO) algorithm was proposed by mimicking the foraging behavior of Escherichia coli in the human esophagus. Artificial shuffled frog leaping algorithm (SFLA) [13] was put forward through the simulation of the frog groups to share information foraging process and exchange mechanism. In [14], Karaboga and Basturk proposed artificial bee colony (ABC) algorithm based on the concept of postpeak, colony breeding, and the way of foraging. But no algorithm is universal; these swarm intelligence optimization algorithms have their own shortcomings such as slow convergence, easy to fall into local optimum, or low accuracy. In [15], the authors proposed an improved ant colony optimization (ICMPACO) algorithm based on the multipopulation strategy, coevolution mechanism, pheromone updating strategy, and pheromone diffusion mechanism in order to balance the convergence speed and solution diversity and improve optimization performance in solving large-scale optimization problem. To overcome the deficiencies of weak local search ability in genetic algorithms (GA) [16] and slow global convergence speed in ant colony optimization (ACO) algorithm in solving complex optimization problems, in [17], the authors introduced the chaotic optimization method, multipopulation collaborative strategy, and adaptive control parameters into the GA and ACO algorithm to propose a genetic and ant colony adaptive collaborative optimization (MGACACO) algorithm for solving complex optimization problems. On the one hand, the ant colony optimization (ACO) algorithm has the characteristics of positive feedback, essential parallelism, and global convergence, but it has the shortcomings of premature convergence and slow convergence speed; on the other hand, the coevolutionary algorithm (CEA) emphasizes the existing interaction among different subpopulations, but it is overly formal and does not form a very strict and unified definition. Therefore, in [18], Huimin et al. proposed a new adaptive coevolutionary ant colony optimization (SCEACO) algorithm based on the complementary advantages and hybrid mechanism.

In 2007, Yang and his coauthors proposed the wolf swarm algorithm [19], which is a new swarm intelligence algorithm. The algorithm simulates the wolf predation process, mainly through the walk, raid, and siege of three kinds of behavior and survival of the fittest population update mechanism to achieve the purpose of solving complex nonlinear optimization problems. Since the wolf pack algorithm is proposed, it is widely used in various fields and has been developed and improved continually, such as follows. In [20], the authors proposed a novel and efficient oppositional wolf pack algorithm to estimate the parameters of Lorenz chaotic system. In [21], the modified wolf pack search algorithm is applied to compute the quasioptimal trajectories for the rotor wing UAVs in the complex three-dimensional (3D) spaces. In [22], wolf algorithm was used to make out polynomial equation roots of the problem accurately and quickly. In [23], a new wolf pack algorithm was designed aiming to get better performance, including new update rule of scout wolf, new concept of siege radius, and new attack step kind. In [24], Qiang and Zhou presented a wolf colony search algorithm based on the leader's strategy. In [25], to explore the problem of parameter optimization for complex nonlinear function, a chaos wolf optimization algorithm (CWOA) with self-adaptive variable step size was proposed. In [26], Mirjalili et al. proposed “grey wolf optimizer” based on the cooperative hunting behaviour of wolves, which can be regarded as a variant of paper [19]; in [27–29], grey wolf optimizer is adopted to solve nonsmooth optimal power flow problems, optimal planning of renewable distributed generation in distribution systems, and optimal reactive power dispatch considering SSSC (static synchronous series compensator).

### 1.2. Motivation and Incitement

From the review of the above literature, the current optimization algorithm of wolf pack follows the principle of “a certain number of scouting wolves lead wolves through greedy search with a specially limited number of times (each wolf has only four opportunities in some literatures),” the principle of “fierce wolves approach the first wolf through a specially limited number of times of rushing” in the call and rushing process, and the principle of “fierce wolves pass through a specially limited number of times of rushing” in the siege process greedy search for prey “in this operation mechanism, the algorithm itself too imitates the actual hunting behavior of wolves, especially in” grey wolf optimization,” which divides the wolves in the algorithm into a more detailed level. The advantage of doing so is that it can effectively guarantee the final convergence of the algorithm because it completely mimics the biological foraging process; however, the goal of the intelligent optimization algorithm of wolves is to solve the optimization problem efficiently, and imitating wolves' foraging behavior is only a way. Therefore, the intelligent optimization algorithm of wolves should be more abstract on the basis of imitating wolves' foraging behavior in order to improve its optimization ability and efficiency. Due to the reasons above, each of variants about wolf pack algorithm mentioned above has its own limitation or inadequacy, including slow convergence, weak optimization accuracy, and narrow scope of application.

### 1.3. Contribution and Paper Organization

To further improve the wolf pack optimization algorithm to make it have better performance, this paper proposed an adaptive shrinking grid search chaotic wolf optimization algorithm using standard deviation updating amount (ASGS-CWOA). The paper consists of five parts including Introduction, Principle of LWPS and CWOA (both of them are classic variants of wolf pack optimization algorithm), Improvement, Steps of ASGS-CWOA, Numerical Experiments, and corresponding analyses.

## 2. Materials and Methods

### 2.1. Principle of LWPS and CWOA

Many variants about original wolf pack algorithm are mentioned above; in this article, we focus on LWPS and CWOA.

#### 2.1.1. LWPS

The leader strategy was employed into traditional wolf pack algorithm, detailed in [24], unlike the simple simulation of wolves' hunting in [26]; LWPS abstracts wolves' hunting activities into five steps. According to the thought of LWPS, the specific steps and related formulas are as follows:

*(1) Initialization*. The step is to disperse wolves to search space or solution space in some way. Here, the number of wolf populations can be represented by *N* and dimensions of the search space can be represented by *D*, and then, we can get the position of the *i*-th wolf by the following equation:

(1) $$\begin{array}{c} x_{i}=\left(x_{i1},x_{i2},\ldots ,x_{id},\ldots ,x_{i\;D}\right),\;\left(i=1,2,\ldots ,N;d=1,2,\ldots ,D\right), \end{array}$$

where *x*_*id*_ is the location of the *i*-th wolf in *d*-th dimension, *N* means the number of wolf population, and *D* is the maximum dimension number of the solution space. Initial location of each wolf can be produced by

(2) $$\begin{array}{c} x_{id}=x_{\min}+\text{rand}\left(0,1\right)\times \left(x_{\max}-x_{\min}\right), \end{array}$$

where rand (0, 1) is a random number distributed uniformly in the interval [0, 1] and *x*_max_ and *x*_min_ are the upper and lower limits of the solution space, respectively.

*(2) Competition for the Leader Wolf*. This step is to find the leader wolf with best fitness value. Firstly, *Q* wolves should be chosen as the candidates, which are the top *Q* of wolf pack according to their fitness. Secondly, each one of the *Q* wolves searches around itself in *D* directions, so a new location can be got by equation (3); if its fitness is better than the current fitness, the current wolf *i* should move to the new location, otherwise do not move, then searching will be continued until the searching time is greater than the maximum number *T*_max_ or the searched location cannot be improved any more. Finally, by comparing the fitness of *Q* wolves, the best wolf can be elected, and it is the leader wolf:

(3) $$\begin{array}{c} x_{i-\text{new}}=x_{id}+\text{rand}\left(-1,1\right)\times \text{step}\_a, \end{array}$$

where step_*a* is the search step size.

*(3) Summon and Raid*. Each of the other wolves will raid toward the location of leader wolf as soon as it receives the summon from leader wolf, and it continues to search for preys during the running road. Finally, a new location can be got by equation (4); if its fitness is better than the one of current location, the wolf will move to the new one, otherwise stay at the current location:

(4) $$\begin{array}{c} x_{id-\text{new}}=x_{id}+\text{rand}\left(-1,1\right)\times \text{step}\_b\times \left(x_{ld}-x_{id}\right), \end{array}$$

where *x*_*ld*_ is the location of the leader wolf in *d*-th dimension and step_*b* is the run step size.

*(4) Siege the Prey [30]*. After the above process, wolves come to the nearby of the leader wolf and will be ready to catch the prey until the prey is got. The new location for anyone except leader wolf can be obtained by the following equation:

(5) $$\begin{array}{c} x_{id-\text{new}}=\left\{\begin{array}{cc} x_{id}, & r_{m}<R_{0},\\ x_{ld}+\text{rand}\left(-1,1\right)\times \text{step}\_\text{c,} & r_{m}>R_{0},r_{m}=R_{0}, \end{array}\right. \end{array}$$

where step_*c* is the siege step size, *r*_*m*_ is a number generated randomly by the function rand (−1, 1) distributed uniformly in the interval [−1, 1], and *R*_0_ is a preset siege threshold.

In the optimization problem, with the current solution getting closer and closer to the theoretical optimal value, the siege step size of wolf pack also decreases with the increase in iteration times so that wolves have greater probability to find better values. The following equation is about the siege step size, which is obtained from [31]:

(6) $$\begin{array}{c} \text{step}\_c=\text{step}\_c_{\min}\times \left(x_{d-\max}-x_{d-\min}\right)\times \;\exp\left(\frac{\ln\left(\text{step}\_c_{\min}/\text{step}\_c_{\max}\right)*t}{T}\right), \end{array}$$

where step_*c*_min_ is the lower limit of the siege step size, *x*_*d*–max_ is the upper limit of search space in *d*-th dimension, *x*_*d*–min_ is the lower limit of search space in *d*-th dimension, step_*c*_max_ is the upper limit of the siege step size, and *t* means the current number of iterations while *T* represents the upper limit one.

Being out of boundary is not allowed to each wolf, so equation (7) is used to deal with the possible transboundary:

(7) $$\begin{array}{c} x_{id-\text{new}}=\left\{\begin{array}{cc} x_{d-\min}, & x_{id-\text{new}}<x_{d-\min},\\ x_{d-\max}, & x_{id-\text{new}}<x_{d-\max}. \end{array}\right. \end{array}$$

*(5) Distribution of Food and Regeneration*. According to the wolf group renewal mechanism of “survival of the strong,” group renewal is carried out including the worst *m* wolves will die and be deleted and new *m* wolves will be generated by equation (2).

#### 2.1.2. CWOA

CWOA develops from the LWPS by introducing the strategy of chaos optimization and adaptive parameters into the traditional wolf pack algorithm. The former utilizes logistic map to generate chaotic variables that are projected to solution space in order to search, while the latter introduces adaptive step size to enhance the performance, and they work well. The equation of logistic map is as follows, which is from [32]:

(8) $$\begin{array}{c} {\text{chaos}}_{k+1}=\mu \times {\text{chaos}}_{k}\times \left(1-{\text{chaso}}_{k}\right){\text{chaos}}_{k}\in \left[0,1\right], \end{array}$$

where *μ* is the control variable and when *μ* is 4, the system is in chaos state.

The strategy of adaptive variable step size search includes the improvement of search step size and siege step-size. In the early stage, wolves should search for preys with a large step size so as to cover the whole solution space as much as possible, while in the later stage, with the wolves gathering continuously, wolves should take a small step size to search for preys so that they can search finely in a small target area. As a result, the possibility of finding the global optimal solution will be increased. And by equation (9), we can get the step size of migration, while by equation (10), the one of siege can be obtained:

(9) $$\begin{array}{c} \text{step}\_a_{\text{new}}=\alpha \times \text{step}\_a0,\;\alpha =1-{\left(\frac{t-1}{2}\right)}^{2}, \end{array}$$

(10) $$\begin{array}{c} \text{step}\_c_{\text{new}}=\text{step}\_c_{0}\times \text{rand}\left(0,1\right)\times \left[1-{\left(\frac{t-1}{2}\right)}^{2}\right], \end{array}$$

where step_*c*_0_ is the starting step size for siege.

### 2.2. Improvement

#### 2.2.1. Strategy of Adaptive Shrinking Grid Search (ASGS)

In the solution space with *D* dimensions, a searching wolf needs to migrate along different directions in order to find preys; during any iteration of the migration, it is according to the thought of the original LWPS and CWOA that there is only a dynamic point around the current location to be generated according to equation (3).

**Figure 1:**
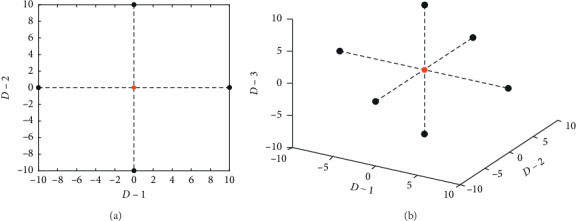
(a) The range of locations of searching or competing wolves in CWOA when dimension is 2; (b) the range of locations of searching or competing wolves in CWOA when dimension is 3, where step_*a* = 10 or step_*c* = 10 (the red point means the current location of some a wolf).

**Figure 2:**
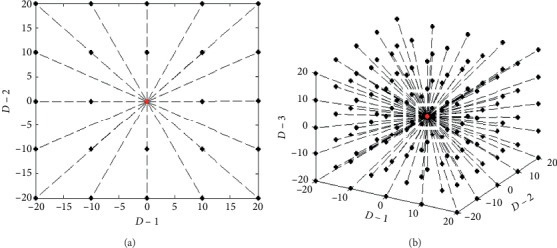
The searching situation of wolves in ASGS-CWOA when dimension is (a) 2 and (b) 3, where step_*a* = 10 or step_*c* = 10 and *K* = 2 (the red point means the current location of some a wolf, and the black ones mean the searching locations of the wolf).

However, a single dynamic point is isolated and not well enough to search in the current domain space of some a wolf; Figures 1(a) and 1(b) show the two-dimensional and the three-dimensional spaces, respectively. In essence, the algorithm needs to take the whole local domain space of the current wolf to be considered in order to find the local best location, so ASGS was used to generate an adaptive grid centered on the current wolf, which is extending along 2 × *D* directions and includes (2 × *K* + 1)^*D*^ nodes, where *K* means the number of nodes taken along any direction. Figures 2(a) and 2(b) show the migration about ASGS in two-dimensional and three-dimensional space, respectively, and for brevity, here, *K* is set to 2, detailed in the following equation:

(11) $$\begin{array}{c} \left[k,x_{id-\text{new}}\right]=x_{id}+\text{step}\_a_{\text{new}}\times k,\;\left(k=-K,-K+1,\ldots ,0,\ldots ,K-1,K\right). \end{array}$$

So a node in the grid can be defined as

(12) $$\begin{array}{c} x_{i-\text{new}}=\left\{\left[k,x_{i1-\text{new}}\right],\left[k,x_{i2-\text{new}}\right],\ldots ,\left[k,x_{i\;D-\text{new}}\right]\right\},\;\left(k=-K,-K+1,\ldots ,0,\ldots ,K-1,K\right). \end{array}$$

During any migration, the node with best fitness in the grid should be selected as new location of the searching wolf, and after any migration, the leader wolf of the population is updated according to the new fitness. It needs to be particularly pointed out that the searching grid will be smaller and smaller as the step_*a*_new_ becomes smaller. Obviously, compared to a single isolated point in traditional methods, the searching accuracy and the possibility of finding the optimal value can be improved by ASGS including (2 × *K* + 1)^*D*^ nodes.

As the same reason, during the process of the siege, the same strategy is used to find the leader wolf in the local domain space including (2 × *K* + 1)^*D*^ points. But the different is that the step size of siege is smaller than the one of migration. After any migration, the leader wolf of the population is updated according to new current fitness, and then, the current best wolf will be the leader wolf temporarily.

#### 2.2.2. Strategy of Opposite-Middle Raid (OMR)

**Figure 3:**
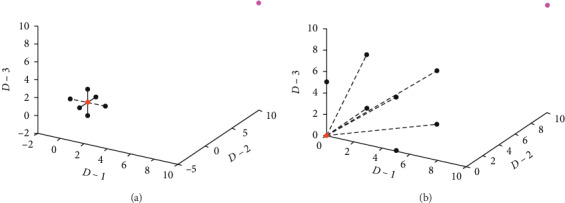
(a) The range of locations of wolves in raid when dimension is 3 according to the original thought of wolf pack algorithm; (b) the range of locations of wolves in raid when dimension is 3 according to OMR, where step_a = 2. The red point means the current position of some wolves, the pink one indicates the position of the lead wolf, and the blue one means the middle point of current wolf and lead wolf.

According to the idea of the traditional wolf pack algorithms, it is unfortunately that raid pace is too small or unreasonable and wolves cannot rapidly appear around the leader wolf when they receive the summon signal from the leader wolf, as shown in Figure 3(a). So OMR is put forward to solve the problem above, and its main clue is that the opposite location of the current location relative to the leader wolf should be calculated by the following equation:

(13) $$\begin{array}{c} x_{id-\text{opposition}}=2\times x_{ld}-x_{id}. \end{array}$$

If the opposite location has better fitness than the current one, the current wolf should move to the opposite one. Otherwise, the following is obtained:

(14) $$\begin{array}{c} \left\{\begin{array}{c} x_{i-m\_d}=\frac{x_{i\_d}+x_{l\_d}}{2},\\ x_{i}=\text{bestfitness}\left(\left[x_{i\_1},x_{i\_2},\ldots x_{i\_\left(D-1\right)},x_{i\_D}\right],\left[x_{i\_1},x_{i\_2},\ldots x_{i\_\left(D-1\right)},x_{m\_D}\right],\ldots \left[x_{i-m\_1},\;x_{i-m\_2},\ldots x_{i-m\_\left(D-1\right)},x_{i-m\_D}\right]\right), \end{array}\right. \end{array}$$

where *x*_*i*−*m*_*d*_ is the middle location in *d*-th dimension between the *i*-th wolf and the leader wolf, *x*_*i*_*d*_ is the location of the wolf *i* in *d*-th dimension, and *x*_*l*_*d*_ is the location of the leader wolf in *d*-th dimension. “Bestfitness” returns a wolf with the best fitness from the given ones.

From equation (14) and Figure 3(b), it is seen that there are 2^*D*^ points among the current point and the middle point, and as the result of each raid, the point with best fitness is chosen as the new location of the current *i*-th wolf. Thereby, not only the wolves can appear around the leader wolf as soon as possible but also they can try to find new preys as far as possible.

#### 2.2.3. Standard Deviation Updating Amount (SDUA)

According to the basic idea of the leader wolf pack algorithm, during the iterations, some wolves with poorer fitness will be continuously eliminated, while the same amount of wolves will be added to the population to make sure that the bad gene can be eliminated and the population diversity of the wolf pack can be ensured, so it is not easy to fall into the local optimal solution and the convergence rate can be improved. However, the amount of wolves that should be eliminated and added to the wolf pack is a fixed number, which is 5 in LWPS and CWOA mentioned before, and that is stiff and unreasonable. In fact, the amount should be a dynamic number to reflect the dynamic situation of the wolf pack during any iteration.

Standard deviation (SD) is a statistical concept, which has been widely used in many fields. Such as in [33], standard deviation was used into industrial equipment to help process the signal of bubble detection in liquid sodium; in [34], standard deviation is used with delay multiply to form a new weighting factor, which is introduced to enhance the contrast of the reconstructed images in medical fields; in [35], based on eight-year-old dental trauma research data, standard deviation was utilized to help analyze the potential of laser Doppler flowmetry; the beam forming performance has a large impact on image quality in ultrasound imaging, to improve image resolution and contrast; in [36], a new adaptive weighting factor for ultrasound imaging called signal mean-to-standard-deviation factor (SMSF) was proposed, based on which researchers put forward an adaptive beam forming method for ultrasound imaging based on the mean-to-standard-deviation factor; in [37], standard deviation was adopted to help analyze when an individual should start social security.

In this paper, we take a concept named “Standard Deviation Updating Amount” to eliminate wolves with poor fitness and dynamically reflect the situation of the wolf pack, which means the population amount of wolf pack and standard deviation about their fitness determine how many wolves will be eliminated and regenerated. Standard deviation can be obtained as follows:

(15) $$\begin{array}{c} \sigma =\sqrt{\frac{1}{N}\sum_{i=1}^{N}{\left(x_{i}-\mu \right)}^{2}}, \end{array}$$

where *N* means the number of wolf pack, *x*_*i*_ means the fitness of the *i*-th wolf, and *μ* is the mean value of the fitness. Then, SDUA is gained by the following formula:

(16) $$\begin{array}{c} \text{SDUA}=\left\{\begin{array}{c} \text{SDUA}+1,\;\text{fitness}\left(xi\right)<\left(\frac{\mu -\sigma }{2}\right),\\ \text{do nothing},\;\text{fitness}\left(xi\right)\geq \left(\frac{\mu -\sigma }{2}\right). \end{array}\right. \end{array}$$

SDUA is zero when the iteration begins; next, difference between the mean value and the SD about the fitness of the wolf pack should be computed, and if the fitness of current wolf is less than the difference, SDUA increases by 1; otherwise, nothing must be done. So the value of SDUA is got, and SDUA wolves should be eliminated and regenerated.

**Figure 4:**
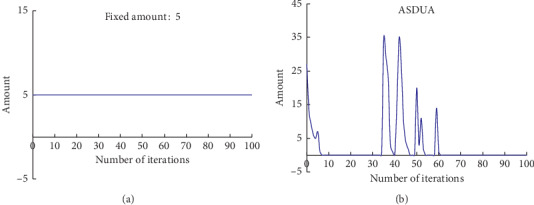
(a) The number that how many wolves will be starved to death and regenerated as the iteration goes on. It is a fixed number 5. (b) The number that how many wolves will be starved to death and regenerated following the new method named SDUA as the iteration goes on.

The effect of using a fixed value is displayed in Figure 4(a), and Figure 4(b) shows the effect of using ASD. It is clear that the amount in the latter is fluctuant as the wolf pack has a different fitness during any iteration and better than the former to reflect the dynamic situations of iterations. Accordingly, not only will the bad gene from the wolf pack be eliminated, but the population diversity of the wolf pack can be also kept better so that it is difficulty to fall into local optimal solution and the convergence rate can be improved as far as possible.

### 2.3. Steps of ASGS-CWOA

**Figure 5:**
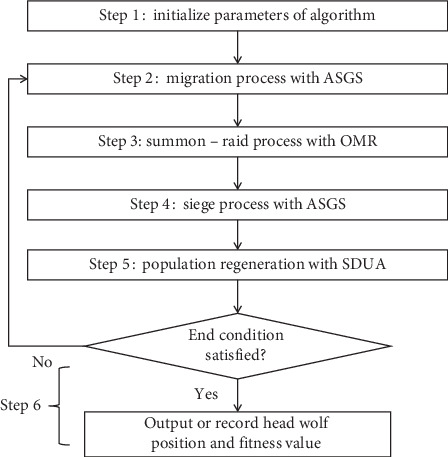
Flow chart for the new proposed algorithm.

Based on the thoughts of adaptive grid search and adaptive standard deviation updating amount mentioned above, ASGS-CWOA is proposed, the implementation steps are following, and its flow chart is shown in Figure 5.

#### 2.3.1. Initialization of Population

The following parameters should be initially assigned: amount of wolf population is *N* and dimension of searching space is *D*; for brevity, the ranges of all dimensions are [range_min_, range_max_]; the upper limit of iterations is *T*, value of step size in migration is step_*a*, value of step size in summon and raid is step_*b*, value of step size in siegement is step_*c*, and the population can be generated initially by the following equation [25]:

(17) $$\begin{array}{c} x_{id}=\text{range}\_\min+\text{chaos}\left(0,1\right)\times \left(\text{range}\_\max-\text{range}\_\min\right), \end{array}$$

where chaos (0, 1) returns a chaotic variable distributed in [0, 1] uniformly.

#### 2.3.2. Migration

Here, an adaptive grid centered on the current wolf can be generated by equation (12), which includes 2 nodes. After migration, the wolf with best fitness can be found as the leader wolf.

#### 2.3.3. Summon and Raid

After migration, the others begin to run toward the location of leader wolf, and during the process of raiding, any wolf keeps searching for preys following equations (13) and (14).

#### 2.3.4. Siege

After summon and raid, all other wolves come to be around the leader wolf in order to siege the prey following equations:

(18) $$\begin{array}{c} \left[k,x_{id-\text{new}}\right]=x_{id}+\text{step}\_c\times k,\;\left(k=-K,-K+1,\ldots ,0,\ldots ,K-1,K\right),\\ x_{i-\text{new}}=\left\{\left[k,x_{i1-\text{new}}\right],\left[k,x_{i2-\text{new}}\right],\ldots ,\left[k,x_{i\;D-\text{new}}\right]\right\}. \end{array}$$

After any siegement, the newest leader wolf can be got, which has best fitness temporarily.

#### 2.3.5. Regeneration

After siege, some wolves with poorer fitness will be eliminated, while the same amount of wolves will be regenerated according to equation (14).

#### 2.3.6. Loop

Here, if *t* (the number of current iteration) is bigger than *T* (the upper limit of iterations), exit the loop. Otherwise, go to 4.2 for loop until *t* is bigger than *T*. And when the loop is over, the leader wolf maybe is the global optima that the algorithm can find.

### 2.4. Numerical Experiments

In this paper, four state-of-the-art optimization algorithms are taken to validate the performance of new algorithm proposed above, detailed in Table 1.

#### 2.4.1. Experiments on Twelve Classical Benchmark Functions

Table 2 shows benchmark functions for testing, and Table 3 shows the numerical experimental data of five algorithms on 12 benchmark functions for testing mentioned above, especially the numerical experiments were done on a computer equipped with Ubuntu 16.04.4 operating system, Intel (R) Core (TM) i7-5930K processor and 64G memory as well as Matlab 2017a. For genetic algorithm, toolbox in Matlab 2017a is utilized for GA experiments; the PSO experiments were implemented by a “PSOt” toolbox for Matlab; LWPS is got from the thought in [24]; CWOA is carried out based on the idea in [25], and the new algorithm ASGS-CWOA is carried out in Matlab 2017a, which is an integrated development environment with M programming language. To prove the good performance of the proposed algorithm, optimization calculations were run for 100 times on any benchmark function for testing as well as any optimization algorithm mentioned above.

Firstly, focusing on Table 3, seen from the term of best value, only new algorithm can find the theoretical global optima of all benchmark functions for testing, so ASGS-CWOA has better performance in optimization accuracy.

**Figure 6:**
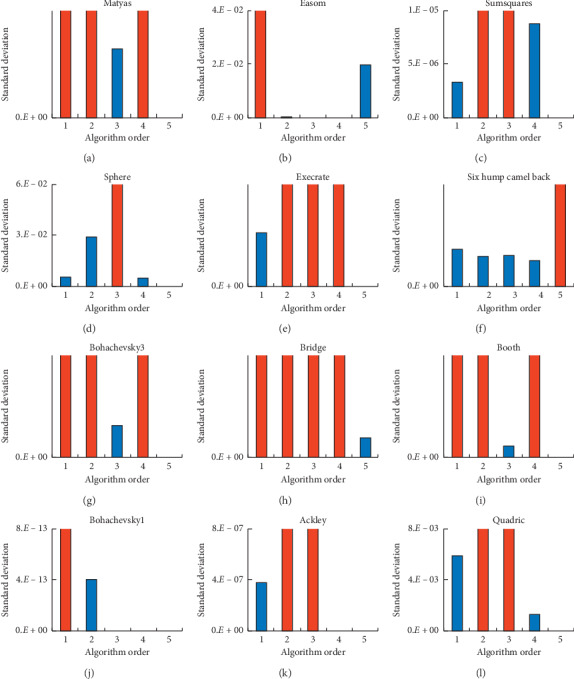
Histograms of the SD about the benchmark functions for testing: (a) –*F*1; (b) –*F*2; (c) –*F*3; (d) –*F*4; (e) –*F*5; (f) –*F*6; (g) –*F*7; (h) –*F*8; (i) –*F*9; (j) –*F*10; (k) –*F*11; (l) –*F*12 (red means the value is out of upper limit of the chart).

Furthermore, for 100 times, the worst and average values of ASGS-CWOA in the all benchmark functions for testing except the *F*2 named “Easom” reach the theoretical values, respectively, and the new algorithm has better standard deviation about best values detailed in Figure 6, from which it can be seen that nearly all the standard deviations are best except *F*2 and *F*6, especially the standard deviations are zero on *F*1, *F*3, *F*4, *F*5, *F*7, *F*9, *F*10, *F*11, and *F*12.  Figure 6(b) shows that the value of standard deviation about ASGS-CWOA on *F*2 is not worst in five algorithms, and it has better performance than GA; Figure 6(f) indicates that the value of standard deviation about ASGS-CWOA on *F*6 reaches 10^−11^, weaker than the values on others; Figure 6(h) demonstrates that the value of standard deviation about ASGS-CWOA on *F*8 reaches 10^−30^, which is not zero, but best in five algorithms. Therefore, ASGS-CWOA has better stability than others.

In addition, focusing on the number of mean iteration, ASGS-CWOA has weaker iteration times on the test function 2 “Easom,” the test function 8 “Bridge,” and the test function 10 “Bohachevsky1,” but it has better performances on the other test functions, especially the iteration times on test function 3, 4, 5, 6, 11, and 12 are 1 or about 1. So ASGS-CWOA has better advantage on performance of iteration number.

**Figure 7:**
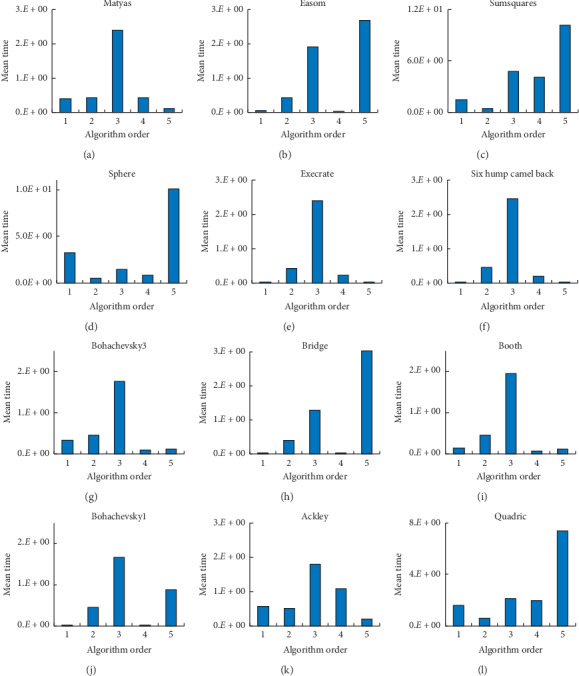
Histograms of the mean time spent on the benchmark functions for testing: (a) –*F*1; (b) –*F*2; (c) –*F*3; (d) –*F*4; (e) –*F*5; (f) –*F*6; (g) –*F*7; (h) –*F*8; (i) –*F*9; (j) –*F*10; (k) –*F*11; (l) –*F*12.

Finally, the mean time spent by ASGS-CWOA are smaller than the others' on *F*1, *F*5, *F*6, and *F*11, as shown in Figures 7(a), 7(e), 7(f), and 7(k), respectively. On *F*7, *F*9, and *F*10, the five algorithms spent time roughly in the same order of magnitude, and ASGS-CWOA has better performance than GA, PSO, and LWPS on *F*7 and *F*9, as shown in Figures 7(g) and 7(i). Unfortunately, ASGS-CWOA spent most time on *F*2, *F*3, *F*4, *F*8, and *F*12, yet it is a comfort that the times spent by ASGS-CWOA are not too much to be unacceptable. This phenomenon conforms to a philosophical truth that nothing is perfect and flawless in the world, and ASGS-CWOA is without exception. Accordingly, in general, ASGS-CWOA has a better speed of convergence. The detailed is shown in Figure 7.

#### 2.4.2. Supplementary Experiments

In order to further verify the performance of the new proposed algorithm, supplementary experiments are conducted on CEC-2014 (IEEE Congress on Evolutionary Computation 2014) testing functions [38], detailed in Table 4. It is different with the above 12 testing functions that CEC2014 testing functions are conducted on a computer equipment with Win7-32 bit, Matlab 2014a, CPU (AMD A6-3400M), and RAM (4.0 GB), due to the match between the given cec14_func.mexw32 and windows 32 bit system, not Linux 64 bit.

**Figure 8:**
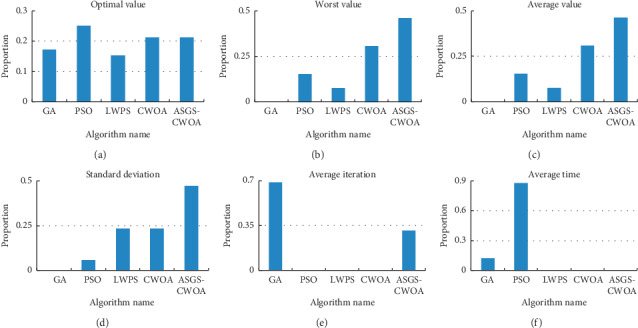
(a) The statistical proportion of each algorithm in the number of best “optimal value” on 16 test functions; (b) the one of best “worst value;” (c) the one of best “average value;” (d) the one of best “standard deviation;” (e) the one of best “average iteration;” (f) the one of best “average time.”

From Table 5 and Figure 8(a), obviously, it can be seen that new proposed algorithm has better performance than GA and LWPS in terms of “optimal value,” which means ASGS-CWOA has better performance in finding the global optima. From Figures 8(b)–8(d), it can be seen that the new proposed algorithm has best performance in terms of “worst value,” “average value,” and “standard deviation;” in other words, the new proposed algorithm has best stability and robustness. From Figure 8(e), the proportion of ASGS-CWOA is better than PSO, LWPS, and CWOA, and it means the new proposed ASGS-CWOA has advantages in time performance.

In a word, ASGS-CWOA has good optimization quality, stability, advantage on performance of iteration number, and speed of convergence.

## 3. Results and Discussion

Theoretical research and experimental results reveal that compared with traditional genetic algorithm, particle swarm optimization, leading wolf pack algorithm, and chaotic wolf optimization algorithm, ASGS-CWOA has better global optimization accuracy, fast convergence speed, and high robustness under the same conditions.

In fact, the strategy of ASGS greatly strengthens the local exploitation power of the original algorithm, which makes it easier for the algorithm to find the global optimum; the strategy of ORM and SDUA effectively enhances the global exploration power, which makes the algorithm not easy to fall into the local optimum and thus easier to find the global optimum.

Focusing on Tables 3 and 5 and Figures 6 and 7 above, compared with the four state-of-the-art algorithms, ASGS-CWOA is effective and efficient in most of terms on benchmark functions for testing, but it has weaker performance in some terms on some benchmark functions for testing, such as functions 2, 3, 4, 8, and 12 shown, respectively, in Figures 7(b)–7(d), 7(h), and 7(l); ASGS-CWOA spent more time on iterations than the four other algorithms. When *D* (dimension of the solution space) is very large, this means too much memory space of the computer is required to implement the algorithm completely according to the formula, and it is impossible to meet the spatial demand growing exponentially, which is also a reflection of its limitations. Moreover, in the supplementary experiments, ASGS-CWOA spent more time than the other algorithms, and its proportion is 0 in the optimal time statistic index of 16 times, detailed in Figure 7(f).

Our future work is to continue perfecting the performance of ASGS-CWOA in all aspects and to apply it to specific projects and test its performance.

## 4. Conclusions

To further improve the speed of convergence, and optimization accuracy under a same condition, this paper proposes an adaptive shrinking grid search chaotic wolf optimization algorithm using standard deviation updating amount. Firstly, ASGS was designed for wolf pack algorithm to enhance its searching capability, through which any wolf can be the leader wolf and this benefits to improve the probability of finding the global optimization. Moreover, OMR is used in the wolf pack algorithm to enhance the convergence speed. In addition, we take a concept named SDUA to eliminate some poorer wolves and regenerate the same amount of wolves, so as to update wolf population and keep its biodiversity.

## Figures and Tables

**Table 1:** Arithmetic configuration.

Order	Name	Configuration
1	Genetic algorithm [16]	The crossover probability is set at 0.7; the mutation probability is set at 0.01, while the generation gap is set at 0.95
2	Particle swarm optimization	Value of individual acceleration: 2, value of weighted value of initial time: 0.9, value of weighted value of convergence time: 0.4, limit individual speed at 20% of the changing range
3	LWPS	Migration step size step*a*: 1.5, raid step size step*b*: 0.9, siege threshold *r*0: 0.2, upper limit of siege step size step*c*_max_ = 10^6^, lower limit of siege step size step*c*_min_ = 10^−2^, updating number of wolves *m*: 5
4	CWOA	Amount of campaign wolves *q*: 5, searching direction *h*: 4, upper limit of search *H*max: 15, migration step size step*a*0: 1.5, raid step size step*b*: 0.9, siege threshold *r*0: 0.2, value of siege step size step*c*0: 1.6, updating amount of wolves *m*: 5
5	ASGS-CWOA	Migration step-size step*a*0: 1.5, upper limit of siege step-size step*c*max = 1*e*6, lower limit of siege step size step cmin = 1*e* − 40; upper limit number of iteration *T*: 600; number of wolf population *N*: 50

**Table 2:** Test functions [25].

Order	Function	Expression	Dimension	Range	Optimum
1	Matyas	*F*1 = 0.26 × (*x*_1_^2^ + *x*_2_^2^) − 0.48 × *x*_1_ × *x*_2_	2	[−10, 10]	min *f* = 0
2	Easom	*F*2 = −cos (*x*1) × cos (*x*2) × exp[− (*x*1 − *π*)^2^ − (*x*2 − *π*)^2^]	2	[−100, 100]	min *f* = −1
3	Sumsquares	*F*3 = ∑_*i*=1_^*D*^*i∗x*_*i*_^2^	10	[−1.5, 1.5]	min *f* = 0
4	Sphere	*F*4 = ∑_*i*=1_^*D*^*x*_*i*_^2^	30	[−1.5, 1.5]	min *f* = 0
5	Eggcrate	*F*5 = *x*_1_^2^ + *x*_2_^2^ + 25 × (sin^2^*x*_1_ + sin^2^*x*_2_)	2	[−*π*, *π*]	min *f* = 0
6	Six hump camel back	*F*6 = 4 × *x*_1_ − 2.1*x*_1_^4^ + (1/3) × *x*_1_^6^ + *x*_1_ × *x*_2_ − 4 × *x*_2_^2^ + 4 × *x*_2_^4^	2	[−5, 5]	min *f* = −1.0316
7	Bohachevsky3	*F*7 = *x*_1_^2^ + 2 × *x*_2_^2^ − 0.3 × cos (3*πx*_1_ + 4*πx*_2_) + 0.3	2	[−100, 100]	min *f* = 0
8	Bridge	*F*8 = $\left(\sin\sqrt{x_{1}^{2}+x_{2}^{2}}\right)/\sqrt{x_{1}^{2}+x_{2}^{2}}$ − 0.7129	2	[−1.5, 1.5]	max *f* = 3.0054
9	Booth	*F*9 = (*x*_1_ + 2 × *x*_2_ − 7)^2^ + (2 × *x*_1_ + *x*_2_ − 5)^2^	2	[−10, 10]	min *f* = 0
10	Bohachevsky1	*F*10 = *x*_1_^2^ + 2 *x*_2_^2^ − 0.3 × cos (3*πx*_1_) − 0.4 × cos (4*π*x_2_) + 0.7	2	[−100, 100]	min *f* = 0
11	Ackley	*F*11 = −20 × exp (−0.2 ×$\sqrt{\left(1/D\right)\sum_{i=1}^{D}x_{i}^{2}}$) − exp ((1/*D*)∑_*i*=1_^*D*^cos 2 πx_*i*_) + 20 + *e*	6	[−1.5, 1.5]	min *f* = 0
12	Quadric	*F*12 = ∑_*i*=1_^*D*^(∑_*k*=1_^*i*^*x*_*k*_)^2^	10	[−1.5, 1.5]	min *f* = 0

**Table 3:** Experimental results.

Function	Order	Best value	Worst value	Average value	Standard deviation	Mean iteration	Average time
*F*1	1	3.64*E* − 12	2.83*E* − 08	3.52*E* − 10	2.83*E* − 09	488	0.4038
	2	4.14*E* − 21	3.26*E* − 14	1.15*E* − 15	3.87*E* − 15	591	0.4282
	3	4.79*E* − 26	4.95*E* − 21	2.58*E* − 22	5.79*E* − 22	585	2.4095
	4	1.04*E* − 119	1.69*E* − 12	5.85*E* − 24	2.13*E* − 13	295	0.4266
	5	**0**	**0**	**0**	**0**	**27.44**	**0.10806**

*F*2	1	−1	−0.00008110	−0.97	0.1714	153	0.0375
	2	−1	−1	−1	3.24*E* − 06	583	0.4196
	3	−1	−1	−1	0	507	1.9111
	4	−1	−1	−1	0	72	0.0321
	5	−1	0	−0.02	0.0196	592.62	2.7036

*F*3	1	8.50*E* − 07	1.61*E* − 05	2.77*E* − 06	3.35*E* − 06	593	1.4396
	2	0.00017021	0.0114	0.0023	0.0022	595	0.4792
	3	7.60*E* − 07	8.7485	1.4802	1.5621	600	4.8319
	4	5.61*E* − 07	1.49*E* − 05	1.07*E* − 06	8.74*E* − 06	600	4.1453
	5	**0**	**0**	**0**	**0**	**1**	10.2205

*F*4	1	0.004	0.0331	0.0127	0.0055	592	3.1902
	2	0.0351	0.1736	0.0902	0.0292	594	0.4868
	3	3.5579	10.3152	8.0497	1.2048	600	1.4742
	4	0.00001114	0.0098	0.0012	0.0048	600	0.8431
	5	**0**	**0**	**0**	**0**	**1**	10.0834

*F*5	1	4.67*E* − 10	4.67*E* − 10	4.67*E* − 10	3.12*E* − 25	73	0.0085
	2	9.20*E* − 22	6.66*E* − 15	1.34*E* − 16	6.78*E* − 16	594	0.4298
	3	1.23*E* − 23	1.76*E* − 19	1.56*E* − 20	2.60*E* − 20	585	2.4112
	4	3.75*E* − 136	1.31*E* − 19	4.28*E* − 22	2.24*E* − 11	195	0.2169
	5	**0**	**0**	**0**	**0**	**1.01**	**1.06** **E** − **03**

*F*6	1	−1.0316	−1.0316	−1.0316	1.79*E* − 15	68	0.0077
	2	−1.0316	−1.0316	−1.0316	1.47*E* − 15	578	0.4597
	3	−1.0316	−1.0316	−1.0316	1.52*E* − 15	519	2.4727
	4	−1.0316	−1.0316	−1.0316	1.25*E* − 15	157	0.1823
	5	−1.0316	−1.0316	−1.0316	5.60*E* − 11	**3.32**	**0.021855**

*F*7	1	4.07*E* − 08	1.64*E* − 06	3.17*E* − 07	5.01*E* − 07	437	0.327
	2	2.78*E* − 16	2.60*E* − 10	8.85*E* − 12	3.10*E* − 11	590	0.4551
	3	0	1.67*E* − 16	7.77*E* − 18	2.50*E* − 17	556	1.7745
	4	0	1.69*E* − 11	6.36*E* − 15	2.36*E* − 11	139	0.0793
	5	**0**	**0**	**0**	**0**	**24.06**	**0.1064**

*F*8	1	3.0054	2.7052	2.9787	0.0629	69	0.0079
	2	3.0054	3.0054	3.0054	4.53*E* − 16	550	0.3946
	3	3.0054	3.0038	3.0053	0.00016125	399	1.2964
	4	3.0054	3.0054	3.0054	9.41*E* − 11	67	0.0307
	5	3.0054	3.0054	3.0054	**9.66** **E** − **30**	600	3.0412

*F*9	1	4.55*E* − 11	3.68*E* − 09	8.91*E* − 11	3.67*E* − 10	271	0.1276
	2	2.47*E* − 20	1.91*E* − 11	1.97*E* − 13	1.91*E* − 12	591	0.4525
	3	6.05*E* − 24	3.12*E* − 19	2.70*E* − 20	5.48*E* − 20	580	1.9606
	4	0	3.15*E* − 11	1.46*E* − 22	5.11*E* − 12	132	0.0738
	5	**0**	**0**	**0**	**0**	**26.86**	**0.11364**

*F*10	1	4.36*E* − 07	0.4699	0.0047	0.047	109	0.0197
	2	0	3.65*E* − 12	1.12*E* − 13	4.03*E* − 13	588	0.4564
	3	0	0	0	0	539	1.6736
	4	0	0	0	0	78	0.025
	5	**0**	**0**	**0**	**0**	184.88	0.86886

*F*11	1	1.5851	1.5851	1.5851	3.78*E* − 07	434	0.5788
	2	1.5934	1.594	1.5935	0.0000826	595	0.4998
	3	1.55*E* − 06	2.1015	0.7618	0.5833	594	1.789
	4	9.21*E* − 07	0.00018069	5.20*E* − 05	4.1549–5	535	1.0822
	5	**0**	**0**	**0**	**0**	**1**	**0.19021**

*F*12	1	2.0399 × *E* − 4	0.0277	0.0056	0.0059	597	1.577
	2	0.0151	0.2574	0.0863	0.0552	592	0.5608
	3	0.139	1.6442	0.9017	0.3516	600	2.1169
	4	4.28*E* − 08	0.0214	0.00038043	0.0013	600	1.9728
	5	**0**	**0**	**0**	**0**	**1**	7.4354

**Table 4:** Part of the CEC'14 test functions [38].

No.	Functions	Dimension	Range	*F*_*i*_^*∗*^ = *F*_*i*_ (*x*∗)
1	Rotated High Conditioned Elliptic Function	2	[−100, 100]	100
2	Rotated Bent Cigar Function	2	[−100, 100]	200
3	Rotated Discuss Function	2	[−100, 100]	300
4	Shifted and Rotated Rosenbrock's Function	2	[−100, 100]	400
5	Shifted and Rotated Ackley's Function	2	[−100, 100]	500
6	Shifted and Rotated Weier Stress Function	2	[−100, 100]	600
7	Shifted and Rotated Griewank's Function	2	[−100, 100]	700
8	Shifted Rastrigin's Function	2	[−100, 100]	800
9	Shifted and Rotated Rastrigin's Function	2	[−100, 100]	900
10	Shifted Schwefel's Function	2	[−100, 100]	1000
11	Shifted and Rotated Schwefel's Function	2	[−100, 100]	1100
12	Shifted and Rotated Katsuura Function	2	[−100, 100]	1200
13	Shifted and Rotated Happycat Function [6]	2	[−100, 100]	1300
14	Shifted and Rotated HGBat Function [6]	2	[−100, 100]	1400
15	Shifted and Rotated Expanded Griewank's Plus Rosenbrock's Function	2	[−100, 100]	1500
16	Shifted and Rotated Expanded Schaffer's *F*6 Function	2	[−100, 100]	1600

**Table 5:** Results of supplementary experiments.

Order	Function	Algorithm	Optimal value	Worst value	Average value	Standard deviation	Average iteration	Average time
1	CEC'14-*F*1	GA	104.9799	30721.11	7855.763	81199210	576.0667	5.9246
		PSO	100	7167.217	966.9491	2364293.9	600	0.18536
		LWPS	104.035	2219.115	650.7181	276101.7	600	8.7827
		CWOA	100.4238	806.3862	303.46	48999.1796	600	9.0239
		ASGS-CWOA	**100**	**100**	**100**	**0**	**17.06**	0.75354

2	CEC'14-*F*2	GA	201.6655	5624.136	1511.7	2202859.8	535.9	5.5281
		PSO	200.0028	5541.629	1877.3888	2559554.7	600	0.1806
		LWPS	208.964	2723.770	769.9574	475101.54	600	9.2476
		CWOA	200.0115	1815.693	489.5193	169111.57	600	8.6269
		ASGS-CWOA	**200**	**200**	**200**	**0**	**15.78**	0.70151

3	CEC'14-*F*3	GA	316.1717	17077.5349	4508.479	23875975	533.3	5.7871
		PSO	300.0007	2391.641	446.9141	101563.53	600	0.18159
		LWPS	300.3407	5796.796	889.49	1104949.7	600	9.2331
		CWOA	300.1111	2196.991	734.5405	232245.81	600	8.5499
		ASGS-CWOA	**300**	**300**	**300**	**0**	**17.62**	0.72644

4	CEC'14-*F*4	GA	400	400.0911	400.0109	0.0005323	86.7333	0.924
		PSO	400	400	400	3.23*E* − 29	600	0.17534
		LWPS	400	400	400	5.90*E* − 17	600	8.7697
		CWOA	400	400	400	0	308.7	3.9497
		ASGS-CWOA	**400**	**400**	**400**	**0**	**12.96**	0.53133

5	CEC'14-*F*5	GA	500	520	507.3432	50.5077	71.4333	0.76291
		PSO	500	515.2678	500.9217	6.7366	600	0.18704
		LWPS	500	500.0006	500.0001	1.49*E* − 08	600	8.8697
		CWOA	500	500	500	1.34*E* − 22	600	8.5232
		ASGS-CWOA	501.7851	520	514.18	28.5606	600	29.8243

6	CEC'14-*F*6	GA	600.0001	600.9784	600.302	0.10694	67.9	0.86812
		PSO	600	600	600	2.86*E* − 11	600	1.3431
		LWPS	600.0001	600.4594	600.0245	0.0087421	600	19.5858
		CWOA	600	600	600	2.32*E* − 25	599.5667	19.1092
		ASGS-CWOA	**600**	**600**	**600**	**0**	**45.16**	17.5578

7	CEC'14-*F*7	GA	700	701.4272	700.3767	0.19181	61.4333	0.67019
		PSO	700	700.6977	700.0213	0.0089771	600	0.18424
		LWPS	700	700.323	700.0795	0.0063773	600	8.5961
		CWOA	700	700.0271	700.0053	4.69*E* − 05	514.5667	7.5231
		ASGS-CWOA	**700**	**700**	**700**	**0**	238.2	11.578

8	CEC'14-*F*8	GA	800	801.9899	801.0945	0.48507	57.6333	0.62438
		PSO	800	801.4806	800.2342	0.13654	594.26	0.17554
		LWPS	800	800.995	800.1327	0.11439	600	8.486
		CWOA	800	801.9899	800.6965	0.53787	497.3667	7.2666
		ASGS-CWOA	**800**	800.995	800.2786	0.19957	469.34	21.8131

9	CEC'14-*F*9	GA	900	903.9798	901.3929	1.1615	58.2667	0.63184
		PSO	900	900.995	900.186	0.10073	591.31	0.17613
		LWPS	900	900.995	900.0995	0.089095	600	8.5478
		CWOA	900	901.9899	900.6965	0.53787	503.5333	6.9673
		ASGS-CWOA	**900**	903.9798	900.5273	0.50398	496.31	23.0867

10	CEC'14-*F*10	GA	1000	1058.504	1005.603	140.4036	63.5333	0.68942
		PSO	1000	1118.750	1009.425	808.3984	591.93	0.19874
		LWPS	1000	1017.069	1000.839	9.1243	600	8.7552
		CWOA	1000	1058.192	1006.533	145.8132	547.8333	7.833
		ASGS-CWOA	1063.306	1363.476	1174.777	7651.0577	600	33.4225

11	CEC'14-*F*11	GA	1100	1333.896	1156.947	3936.7124	62.2333	0.67663
		PSO	1100	1218.438	1107.871	228.3351	593.69	0.19941
		LWPS	1100	1100.624	1100.208	0.047643	600	9.0343
		CWOA	1100	1218.438	1105.273	459.2835	534.2	7.6136
		ASGS-CWOA	1100.312	1403.453	1323.981	5000.5589	600	29.089

12	CEC'14-*F*12	GA	1200.000	1205.058	1200.198	0.84017	74.1667	0.90516
		PSO	1200	1200.286	1200.008	0.00091935	590.03	1.0801
		LWPS	1200.001	1201.136	1200.242	0.041088	600	17.2774
		CWOA	1200	1200	1200	1.49*E* − 20	600	17.6222
		ASGS-CWOA	**1200**	1200.016	1200.001	9.25*E* − 06	537.61	173.256

13	CEC'14-*F*13	GA	1300.005	1300.188	1300.02	0.0012674	69.2667	0.74406
		PSO	1300.003	1300.303	1300.123	0.0073239	591.87	0.18376
		LWPS	1300.007	1300.267	1300.089	0.0039127	600	8.7117
		CWOA	1300.000	1300.132	1300.017	0.0010414	600	9.0362
		ASGS-CWOA	1300.013	1300.242	1300.120	0.003075	600	28.6266

14	CEC'14-*F*14	GA	1400.000	1400.499	1400.264	0.035262	57.9	0.63369
		PSO	1400	1400.067	1400.023	0.00039174	591.8	0.19591
		LWPS	1400.000	1400.175	1400.037	0.0015846	600	9.0429
		CWOA	1400.000	1400.179	1400.030	0.001558	600	9.3025
		ASGS-CWOA	1400.111	1400.491	1400.457	0.0032626	600	30.4334

15	CEC'14-*F*15	GA	1500	1500.175	1500.073	0.0033323	54.7667	0.59602
		PSO	1500	1500.098	1500.012	0.0004289	591.32	0.18265
		LWPS	1500	1500.139	1500.021	0.0010981	600	7.4239
		CWOA	1500	1500.098	1500.015	0.0003538	477.6667	6.5924
		ASGS-CWOA	**1500**	**1500.020**	**1500.001**	**2.24E** − **05**	101.29	4.8

16	CEC'14-*F*16	GA	1600	1600.746	1600.281	0.038563	53.8333	0.58607
		PSO	1600	1600.356	1600.015	0.001519	593.95	0.18491
		LWPS	1600.019	1600.074	1600.024	0.000272	600	8.7907
		CWOA	1600	1600.254	1600.058	0.0031938	593.5	9.0235
		ASGS-CWOA	**1600**	**1600.019**	**1600.007**	**8.69E** − **05**	545.55	26.1972

## Data Availability

The tasks in this paper are listed in https://pan.baidu.com/s/1Df_9D04DSKZI2xhUDg8wgQ (password: 3fie).
